# Editorial

**DOI:** 10.1155/S1064744901000011

**Published:** 2001

**Authors:** Sebastian Faro


### Editorial

*Infectious Diseases in Obstetrics and Gynecology* has embarked on a new era with our new publishers Parthenon Publishing. This is a major step in the journal's development since it is now part of a publishing company with a long list of publications, and a tradition of publishing material devoted to obstetrics and gynecology, as well as other health issues pertaining to women.

*Infectious Diseases in Obstetrics and Gynecology* will maintain its current cover and colors, but other changes have already been made. Beginning with Volume 9 in 2001, the journal will be a quarterly publication. This should enable us to provide our readers with more substantive issues that cover more infectious disease-related topics. In 2001 the journal also plans to introduce new sections in an effort to promote reader participation. A main feature on immunizations is planned, as well as a case presentation section that invites readers to submit interesting cases for discussion. We also hope to develop a section that focuses on the presentation, diagnosis, and management recommendations of specific subjects for discussion from a clinical point of view. Finally, I will discuss a specific article accepted for publication in each issue.

Together with the publisher, it is my hope that this journal will become a dynamic publication that gives its readers the opportunity to gain insight into the latest research developments, and also to increase their fund of knowledge in infectious diseases in obstetrics and gynecology. The journal will also serve as a reference for the management of infectious diseases in obstetrics and gynecology by providing the reader with the latest in infectious disease diagnosis, management, and treatment regimens, as well as presenting references for a detailed discussion on the subject. Thus, the journal will be a current and updated source for infectious diseases in obstetrics and gynecology.

Sebastian Faro, MD, PhD
Editor-in-Chief

